# A Case of Amputation of the Femur, for Mortificaton of the Leg, with Two Cases Catalytic Disease, Treated with the Sulphites of Soda and Lime

**Published:** 1863

**Authors:** A. Fisher

**Affiliations:** Chicago


### CHICAGO MEDICAL EXAMINER.

N. S. DAVIS, M.D., Editor.

VOL. IV. MARCH AND APRIL, 1863. NO. 3.

### Original Contributions.

ARTICLE VIII.

### A CASE OF AMPUTATION OF THE FEMUR FOR MORTIFICATION OF THE LEG, WITH TWO CASES CATALYTIC DISEASE, TREATED WITH THE SULPHITES OF SODA AND LIME.

By A. FISHER, M.D., of Chicago.

Read before the Chicago Medical Society, March 27, 1863.

Mrs. McIntosh, a native of Netherlands, aged 33, of a sanguine and nervous temperament, strong and robust constitution, says: that she has always enjoyed good health until last January, when she had scarlet fever, and before fully recovering from it, she began to have severe pain in the left limb with a high fever, which continued to grow worse until the limb was mortified.

I first saw her Saturday, January 31st. I found the left limb destroyed by sphacelus to within about four inches of the knee-joint, with a well-marked line separating the dead portion from the living. The thigh was much enlarged and tender along the track of the femoral vessels; tongue red, smooth, and dry; pulse 120 and weak; bowels loose; no appetite, and takes very little nourishment. She says that no physician has seen her for the past six days, and that during that time she took no medicine, although a woman had been applying plasters to the limb, without any benefit.

Being unable to obtain sufficient information to give the previous symptoms and treatment of the case in detail, I shall say nothing about it, for fear that I might state it incorrectly. She says, however, that mustard was at first applied to the limb, then tinc. of iodine, and, afterwards, a poultice made of yeast and shorts. Prescribed quinine and camphor aa grs. jj. with opium gr. a-quarter every four hours; promised to amputate the limb on Monday, if she was in a proper condition.

Monday, February 2d.—Found her about the same. The wound smells very bad; says that she wants the limb amputated. About 11 o'clock, A.M., Dr. Wickersham administered chloroform to the patient, before removing her from the bed. We then placed her upon the table and applied a tourniquet. The artery, as it passes under Poupart's ligament on the effected side, could with difficulty be felt, whilst it was quite strong on the other side. The limb was severed by the flap-operation, above and as near the knee as possible, and have integuments enough to form a flap; in presence of Drs. Marguerat, Heydock, and Smith, who assisted me. Dr. Wickersham attending the patient, keeping her in a proper condition. There was very little hemorrhage, three or four small arteries only were taken up and tied; not a drop of blood was found in the femoral artery, and it was so contracted and filled with pus that we did not ligate it. The femoral vein had the appearance of being injected with red wax. The flaps were then brought together and confined with sutures; adhesive-plasters and the usual dressings were then applied, and the patient returned to her bed without being conscious of the operation. Half gr. morphia was given in some brandy as soon as she aroused sufficiently to take it. 9, P.M.—Pulse 110, full and strong; complains of some pain in the stump; ℞. morphia gr. $\frac{1}{4}$, every four or six hours, if necessary, to allay pain.

3d.—Pulse 120, strong and hard; tongue dry, smooth, and red; stump badly swollen, measures twenty-three inches in circumference, and tender over the vessels; temperature normal; takes very little nourishment. ℞. chlorate potass gr. x. in solution every six hours, morphia gr. ¼ every six hours.

4th.—Pulse 118, rather hard and thready; tongue still dry, red, and smooth; stump rather more swollen and tender; inclined to be stupid; bowels moved two or three times. ℞. sulphite soda in solution ʒj. every six hours, turpentine emulsion with tinc. opii. every six hours.

5th.—Pulse 116, softer and fuller; feels and look more cheerful; tongue about the same; stump not quite as hard; bowels moved three times. Diet.—Milk and beef-tea, and crust water for drinks. Continue same medicines.

6th.—About the same in all respects; dressed the stump, it looks well and begins to suppurate, it is not quite as hard or as much swollen. Continue same medicines, with wine if she wants it.

7th.—Rather more comfortable; continue same course.

9th.—Pulse 100, full and regular; tongue more moist, and not as red; bowels rather loose; dressed the stump, it suppurates profusely; pus rather thin, but color good; edges of the wound look healthy and begin to unite; swelling of the stump subsiding. Continue the sulphite soda and emulsion, with Maderia wine.

12th.—Is improving gradually; pulse 96, full and soft; tongue moist and clean; stump looks well and is healing by granulation, suppurates profusely; pus laudable; has some appetite, takes oysters and more nourishment. Continue sulphite soda, and give tinc. muriate of iron and strychnine, instead of the turpentine emulsion.

14th.—Has fever nights; pulse 100; tongue not quite as moist, otherwise, about the same. Continue the tinc. muriate of iron and sulphate quinine gr. iiij. every six hours, and omit the sulphite soda.

17th.—Fever at nights has subsided; pulse 96; tongue rather dry, complains of thirst; has not much relish for food. ℞. sulphite soda ʒj. every six hours, omit the quinine and continue tinc. muriate of iron.

19th.—Pulse 94, soft, full, and regular; thirst abated,—

tongue not as dry; wound secretes laudable pus and looks well, —it is now more than half united, ligatures have all been removed; she takes broth and beef-steak with a relish; bowels quite loose, though the discharges are consistent. Continue same treatment.

22d.—Pulse 85; tongue moist and clean; the wound healing by granulation, and looks healthy. Continue same medicines.

March 1st.—Feels well; pulse 85; appetite good; bowels regular; wound nearly healed; some induration in the traek of the large vessels, though not tender on pressure. Follow same course.

7th.—Pulse 80; tongue clean; appetite good; bowels move once daily; swelling of the stump subsiding,—wound nearly healed; and she is gaining strength rapidly. ℞. no medicine.

14th.—Has been improving steadily; appetite good; wound nearly healed. Gave no medicine.

24th.—Found her sitting up in bed quite well, though weak; wound entirely healed, with as good a stump for an artificial limb as I ever saw.

I will defer making any comments on the case until I have given the notes of two other cases, which, I believe originated from the one just related, as they are man and wife, and were both sick at the same time. I will merely copy my notes as taken at the time of my visits.

I was called, Feb. 26th, to see Mr. Rademaker, a healthy robust man, about 40 years old, who had received an injury of the ankle and a slight cut on the patella. I advised cloths wet in a solution of salt and vinegar to be applied to the ankle, and drew a strip of adhesive-plaster over the wound, with cloths wet in cold water over the knee.

Mrs. R., his wife, (a relative of Mrs. McIntosh, who had been nursing and taking care of her for three weeks, or more, after the amputation,) complained of pains in the joints, loss of appetite, &c., but did not wish me to prescribe for her.

March 1st.—Saw them again: Mr. R. complained of soreness about the wound on the patella; it looked red, and was quite tender for two or three inches around the wound. ℞. cranberry poultice over the inflamed part. Mrs. R. was no better. Her bowels being constipated, I prescribed a mild cathartic.

4th.—Mr. R. has erysipelatous inflammation on the external and anterior portion of the limb, extending from the knee nearly to the hip, which is very painful and tender on pressure. Continue same poultice. Mrs. R. not as well, complains of pain in all the joints, has fever and chills, no appetite, pulse 110, tongue coated, bowels loose. ℞. tincture of cimicifuga, ʒss. every four hours, with morphia gr. $\frac{1}{4}$, every four or six hours if she has severe pain.

7th.—Mr. R. complains of severe pain of the limb above the knee, which is much swollen, and tender on pressure, with evident signs of suppuration. Ordered slippery elm poultice. Mrs. R. is very much worse, her countenance is sunken, and she looks cadaverous; pulse 120, small and quick; tongue dry and coated; severe pain in all the joints; and she is unable to move even the fingers; with a blush of red spots over the joints of the fingers, toes, ankles, and other parts of the body; bowels loose, moving twelve or fifteen times in the twenty-four hours; has no appetite and takes no nourishment. Ordered sulphite of lime, ʒj. every four hours, with a pill of plumbi acetas gr. ij. and opii. pulv. gr. ss. as often as necessary to control the bowels.

March 8th.—Mr. R.'s limb is very much swollen, with evident fluctuation; made an opening in the popliteal region, and let out, at least, a pint of pus, which relieved him very much. He never had any severe constitutional symptoms more than would be expected from the local disease, therefore, I gave him no medicine. Mrs. R. appears rather better; pulse 110; not quite as much pain; bowels move six or eight times in the twenty-four hours. Continue same course.

9th.—Mr. R. is very much better,—has no pain; swelling nearly gone, though the redness on the surface continues on the upper part of the thigh. Mrs. R. is improving; pulse 100, more soft and full; tongue not as dry; bowels moved three or four times daily; pain less severe; can move the fingers better; takes some nourishment for the first time for several days. Continue the sulphite lime, and pills if necessary.

12th.—Mr. R. is gaining fast,—sits up and eats as usual, though the abscess discharges slightly. Mrs. R. is improving rapidly also; pulse 85, soft and full; tongue looks better; pain and redness of the joints subsiding. Continue the sulphite lime and omit the pill.

16th.—Mr. R. not as well; pulse 100, small and quick; tongue coated; inflammation and swelling about the hip, which is quite tender on pressure, with signs of suppuration. ℞.—sulphite lime, ʒj. every four hours, and apply slippery elm poultice. Mrs. R. still improving; pulse 75; tongue moist and cleaning; appetite better; pain and stiffness in the joints relieved, with the exception of the left wrist; bowels regular, without any opiate. Continue sulphite lime the same.

18th.—Mr. R. is much better; pulse 90, and more full; tongue more moist; swelling and redness about the hip less. He says: that he began to improve in less than twenty-four hours after taking the sulphite of lime. Mrs. R. is still doing well, though she has some pain in the left wrist. Continue the sulphite of lime.

21st.—Mr. R. continues to improve; pulse 80; tongue moist; appetite returned; very little redness or swelling of the hip. Continue the same. Mrs. R. is very much better; pulse 65, full and soft; tongue not much coated and moist; some appetite. Continue sulphite lime every six hours, instead of every four hours, and give tinc. muriate of iron every six hours.

24th.—Found them both sitting up, and so well that I discontinued my visits; but advised them to continue taking the medicines a few days longer.

*Remarks.*—The sulphites of lime and soda having never been used internally as remedial agents to any extent, and in catalytic diseases never before to my knowledge, I deem it prudent to state how I came to prescribe them:—My attention was directed to their use in these cases by reading the review of an article on "Diseases depending upon morbific fermentation and their treatment," by Dr. G. Polli, of Milan; published in part 46 of *Braithwait's Retrospect*, and taken from the *Dublin Quarterly Journal*, May, 1862, page 367.

From the well-known power of sulphurous acid in preventing fermentation and decomposition of organic substances, Dr. POLLI made numerous experiments on living dogs, with a view of ascertaining whether the sulphurous acid in combination with the alkalies or alkaline earths, might not be introduced into the system of the living animal, without being changed into sulphuric acid or the sulphates, and if so, whether they would not act beneficially in preventing or curing catalytic diseases, or those diseases supposed to be caused by morbific ferments or blood-poisoning. His experiments were numerous,—sixty-eight in number,—carefully conducted, and appear to me to be conclusive in establishing the following results:—

With a view of determining the quantity of the sulphites of soda, potassa, magnesia, or lime that could be safely administered to a dog, he gave to one, weighing eight kilogrammes, ten grammes of these salts daily for fifteen successive days, and, afterwards, gave to a dog of about the same weight fifteen grammes at a dose. During the experiment, he killed several of these dogs, and, upon examination, found the stomach and intestines in a perfectly normal condition. Having proved that the sulphites could be given in large quantities with impunity, he then experiments to ascertain whether they were conveyed through the system in the form of sulphites, or whether they become oxydized and formed sulphates. He gave the sulphite of soda to one dog, the sulphite of magnesia to another, and to the third he gave the same food but no sulphites. The dogs were put to death at the same time. In the first two, the sulphites were detected in the urine, blood, and every part of the system; in the third, no traces of sulphurous acid could be found, showing that large quantities of the sulphites can be administered without producing any deleterious effects, and that they remain in the system for some time without being changed to sulphates.

After injecting pus and putrid substances into the femoral veins of dogs, he says:—Experiments carefully practiced on animals have given the following results:—

1st. "That the injection of a certain quantity of pus into the circulation, produces pyema, and such diseases as are characterized by multiple abscesses.

2nd. "That the injection of putrid matter produces septicemia, or those diseases recognized by the name of putrid, infectious, and which are characterized by typhoid gastro-enterites.

3rd. "That the injection of matter obtained from contagious diseases, glanders, for instance, will re-produce the same affections."

After relating numerous experiments of injecting pus and putrid substances into the veins of dogs, and giving them the sulphites of soda, lime, etc., as antidotes, the Reviewer says: "Several dogs were prepared with different quantities of sulphites, for several days previous to being injected with putrid pus; the result was, that those which had received the smaller doses of sulphites died, while those which had been liberally supplied with them recovered, and the rule seemed to be pretty constant, that the more pus was injected, the greater quantity of sulphites was required to antagonise it. Several dogs were injected with putrid blood; they all, with one exception, died. Other dogs, prepared with the sulphites and then injected with the same blood, all recovered, as well as some dogs who were injected with putrid blood, diluted with twice its bulk of solution of bi-sulphite of soda. A number of dogs were similarly treated with the discharge collected from a glandered horse, and the result was the same."

The *Reviewer* further says: "In conclusion we would say, that if the author has not deceived himself in his experiments, nor overrated the value of these substances, but has really discovered in the sulphites a remedy for catalytic diseases, and a prophylactic against them, he has conferred a boon as great, or perhaps even greater, than did JENNER, by his great discovery of vaccination."

After carefully reading the article above alluded to, I concluded that if Dr. POLLI (known to be a scientific physician,) was not mistaken in his conclusions, from his experiments, the sulphites might be given, without any risk of doing harm, with the well grounded hope that they would be invaluable, in the large class of diseases called catalytic; if they operated the same on the human system as on the dogs upon which he experimented.

That the cases which I have just related belong to the class of diseases which Dr. POLLI calls catalytic, there can be no doubt. In fact, the morbid specimen of a portion of the popliteal artery and vein, which I here exhibit, shows, that there must have been inflammation of both of those vessels. For the artery is contracted, filled with pus and shreds of fibrine, adherent to its internal coat, which is uneven and rough. The vein, as you will see, is completely filled with a hard, redish substance, the product of inflammation of that vessel.

Arterites is a rare disease, and when complicated with phlebitis, must be rare indeed. The inflammation of the blood-vessels, in the case of Mrs. M., was undoubtedly the sequelæ of blood-poison of scarlet fever. I have long been of the opinion that phlebitis occurs as a sequence of that disease, more frequently than is generally supposed. In fact, we know, that ulcerations and abscesses, with a low grade of fever, which are kin to phlebitis, often follow scarlatina, and are caused by the same morbific blood-poison that generates phlebitis, consequently we may expect it to occur, in cases where the poison is sufficiently concentrated.

I am generally far from being the first to prescribe new remedies, having so frequently seen them overrated at first, I usually defer prescribing them until they have been fairly tested by those who can experiment on a large scale, and ascertain their true value; but soon after the amputation, Dr. BARTLET showed me the review of the article by Dr. POLLI, and after perusing it with great care, I thought, in the case of Mrs. McIntosh, we had one in point, and having but little confidence in the usual remedies in her case, I concluded to try the sulphite of soda, and carefully watch its effect. Though in her forlorn condition, I did not deem it prudent to trust to it alone, and as other remedies were given at the same time, we cannot say, with certainty, that the sulphite of soda was of any special benefit. One thing, however, is certain, that she improved while taking it, and grew worse when it was omitted, as you will see by the symptoms and treatment, which were recorded with great care at every visit.

The case of Mrs. Rademan, I at first diagnosed rheumatism, or a disease of that character, and prescribed accordingly, but in a day or two, as the disease became more fully developed, I became satisfied that it was of the catalytic order of diseases, and that it was propagated from Mrs. McIntosh, when Mrs. R. was nursing her. If you recollect, Mrs. R. grew worse fast, while taking the tincture of cimicifuga, and I considered her in a very dangerous condition when we commenced with the sulphite of lime, March the 7th. From that time we continued the sulphite of lime, in drachm doses every four hours, until the 21st, when she became convalescent. Between the 7th and 21st of March she took 10½ ounces of it, and nothing else in the form of medicines, with the exception of the pill of sugar of lead and opii. to restrain the discharges from the bowels. Now in this case we know, that if Mrs. R. was cured by any medicine, it must have been the sulphite of lime, for she took no other medicine, with the exception of the plumbi acetas and opii. pill, and that was discontinued when the diarrhœa was controlled, and she continued to improve the same, or even more rapidly, after the pill was omitted; to be sure, after the 21st, she took, with the sulphite lime, tinc. ferri. murias and strychnine, each every six hours as a tonic; but if you remember, she was entirely relieved before taking the iron and strychnine,—nothing but debility remaining.

In regard to the case of Mr. Rademan, I feel confident that his wound was innoculated in some way from his wife, Mrs. R., perhaps, before I saw him. At first, it was a local phlegmonous erysipelas, and after the abscess was opened he appeared well, and I prescribed no remedies. But in a few days the parts about the hip became inflamed, and a new abscess appeared to be forming, with severe constitutional symptoms. I then gave him sulphite of lime in drachm doses every four hours, with no other medicine; and in less than forty-eight hours he was much better, the local inflammation disappearing, as well as the fever and constitutional symptoms. He felt assured that the sulphite of lime was what relieved him.

Since the above article was written, I have prescribed the sulphites of soda and lime in four or five well-marked cases of catalytic or blood-poison diseases, with the happiest effect. In every instance there has been a marked improvement in less than forty-eight hours after commencing its use, and in no case has it been detrimental, to my knowledge. I have notes of the cases, and will at some future time report them.

With regard to the dose of the sulphites of soda and lime, I can only say, that I commenced with ʒj. every four or six hours; calculating from the quantity which Dr. POLLI gave the dogs, that it would be a medium or rather a small dose for an adult, and the effect was so good that I did not think best to increase or diminish it; however, in very bad cases, I think an ounce or more in the twenty-four hours might be given to advantage. I would further say, that the sulphites of soda and lime, which I have prescribed, were pure, prepared by Dr. SQUIBBS for internal use, and not the common article kept by druggists for artists, &c.

